# The ubiquitous ‘cancer mutational signature’ 5 occurs specifically in cancers with deleted *FHIT* alleles

**DOI:** 10.18632/oncotarget.22321

**Published:** 2017-11-06

**Authors:** Stefano Volinia, Teresa Druck, Carolyn A. Paisie, Morgan S. Schrock, Kay Huebner

**Affiliations:** ^1^ Department of Morphology, Surgery & Experimental Medicine, University of Ferrara, 44121 Ferrara, Italy; ^2^ Department of Cancer Biology & Genetics, The Ohio State University Comprehensive Cancer Center & Wexner Medical Center, Biomedical Research Tower, Columbus, OH 43210, USA; ^3^ University of Washington, Department of Biomedical Informatics & Medical Education, Center for Infectious Disease Research, Seattle, WA 98109, USA

**Keywords:** exome sequences, mutational signatures, data mining, clock-like signatures, cancer genes

## Abstract

The *FHIT* gene is located at the fragile FRA3B locus where activation by carcinogen-induced and endogenous replication stress causes *FHIT* deletions even in normal cells over a lifetime. Our lab has shown that loss of *FHIT* expression causes genome instability and provides single-strand DNA substrates for APOBEC3B hypermutation, in line with evidence that *FHIT* locus deletions occur in many cancers. Based on these biological features, we hypothesized that *FHIT* loss drives development of COSMIC mutational signature 5 and here provide evidence, including data mining of >6,500 TCGA samples, that *FHIT* is the cancer-associated gene with copy number alterations correlating most significantly with signature 5 mutation rate. In addition, tissues of Fhit-deficient mice exhibit a mutational signature strongly resembling signature 5 (cosine similarity value = 0.89). We conclude that *FHIT* loss is a molecular determinant for signature 5 mutations, which occur in all cancer types early in cancer development, are clock-like, and accelerated by carcinogen exposure. Loss of *FHIT* caretaker function may be a predictive and preventive marker for cancer development.

## INTRODUCTION

Frequent deletions within the fragile FRA3B/*FHIT* locus in preneoplasias [1–6], leading to loss or reduction of Fhit protein expression, are due to the sensitivity of this common fragile site to replication stress. In normal, transformed, and cancer-derived cell lines, Fhit-depletion causes replication stress-induced DNA double-strand breaks [7, 8] and defects in replication fork progression, through down-regulation of *Thymidine Kinase 1* (TK1) expression and reduced thymidine triphosphate pool levels; thymidine supplementation rescues DNA replication defects and suppresses DNA breakage in Fhit-deficient cells. Depletion of Fhit does not activate the DNA damage response, allowing continued cell proliferation and ongoing chromosomal instability [7]. Also, Waters et al [9] showed that *FHIT*-low/APOBEC3B(A3B)-high cytidine deaminase-expressing lung adenocarcinomas displayed increased numbers of A3B signature mutations, while tumors with normal *FHIT* expression did not exhibit A3B hypermutation, in spite of high A3B expression; thus, A3B overexpression and Fhit-loss induced DNA damage are independent events that when occurring together, result in increased A3B induced mutations. These biological and genetic features of cells and cancers with reduced *FHIT* expression, suggested that reduced *FHIT* expression might drive generation of a specific cancer-associated 'mutational signature' defined by Alexandrov et al [10] as Catalog of Somatic Mutations in Cancer (COSMIC; http://cancer.sanger.ac.uk/cosmic) [11] mutational signature 5.

Using a 96-category single base substitution (SBS) classification, based on type of substitution and bases immediately 5′ and 3' to the mutated base, Alexandrov & colleagues have identified 30 distinct mutational signatures across 40 cancer types, accessible in the COSMIC database [10, 11]. Some signatures, such as signature 5, are present in multiple cancer types, while others are restricted to a certain class of cancer. For instance, signature 7, which is found primarily in skin cancers and is characterized by the presence of CC>TT dinucleotide mutations at dipyrimidines, is believed to be caused by Ultraviolet light [11]. In follow-up studies [12, 13], Alexandrov & coauthors used mutations from >10,000 cancer genomes representing 36 cancer types, to investigate clock-like mutational processes in human cells and reported that only two mutational signatures showed clock-like properties, with different mutation rates in different tissues [12, 14]. Since the mutation rates for the two signatures were not correlated, it was concluded that processes driving signatures 1 & 5 throughout life, were different but mutation numbers for both increased in correlation with age. Thus, the set of 'somatic mutations shared by most members of a cancer cell population, is the set that was present in the progenitor cell of the final dominant clonal expansion of the cancer' [14]. Since the *FHIT* gene encompasses a common fragile site, common to all humans (and mice), the locus accumulates chromosome gaps in some cells and likely most tissues throughout life [15–17]; also age-associated mutation would increase due to loss of genome caretaker function in the cells with *FHIT* locus gaps/deletions. That is, endogenous replication stress associated with aging results in alterations within the FRA3B locus, loss of *FHIT* genome caretaker function, imbalance of deoxynucleotide triphosphate pools and enhanced replication stress [7, 8]. We have thus proposed that loss of *FHIT* expression underlies development of the ubiquitous signature 5 mutations in human cancers.

## RESULTS

### The mutation profile of *Fhit* knockout mouse genomes resembles COSMIC signature 5

Allele copy number alterations (CNAs) and expression changes are observed in Fhit-deficient cells in conjunction with alterations in cell proliferation and exome mutations [16, 18–20]. To define genomic changes associated with preneoplastic changes *in vivo*, exome DNAs were sequenced for mouse *Fhit* wild-type (wt) and knockout (ko) tissues and established kidney cell lines. The ko exome DNAs showed increased frequencies of small insertions, deletions and SBSs relative to wt DNAs, some related to preneoplastic changes [7, 18–20]. Thus, *Fhit* loss provided a ‘mutator’ phenotype, a cellular environment in which mild genome instability permits clonal expansion through proliferative and survival advantage.

**Figure 1:**
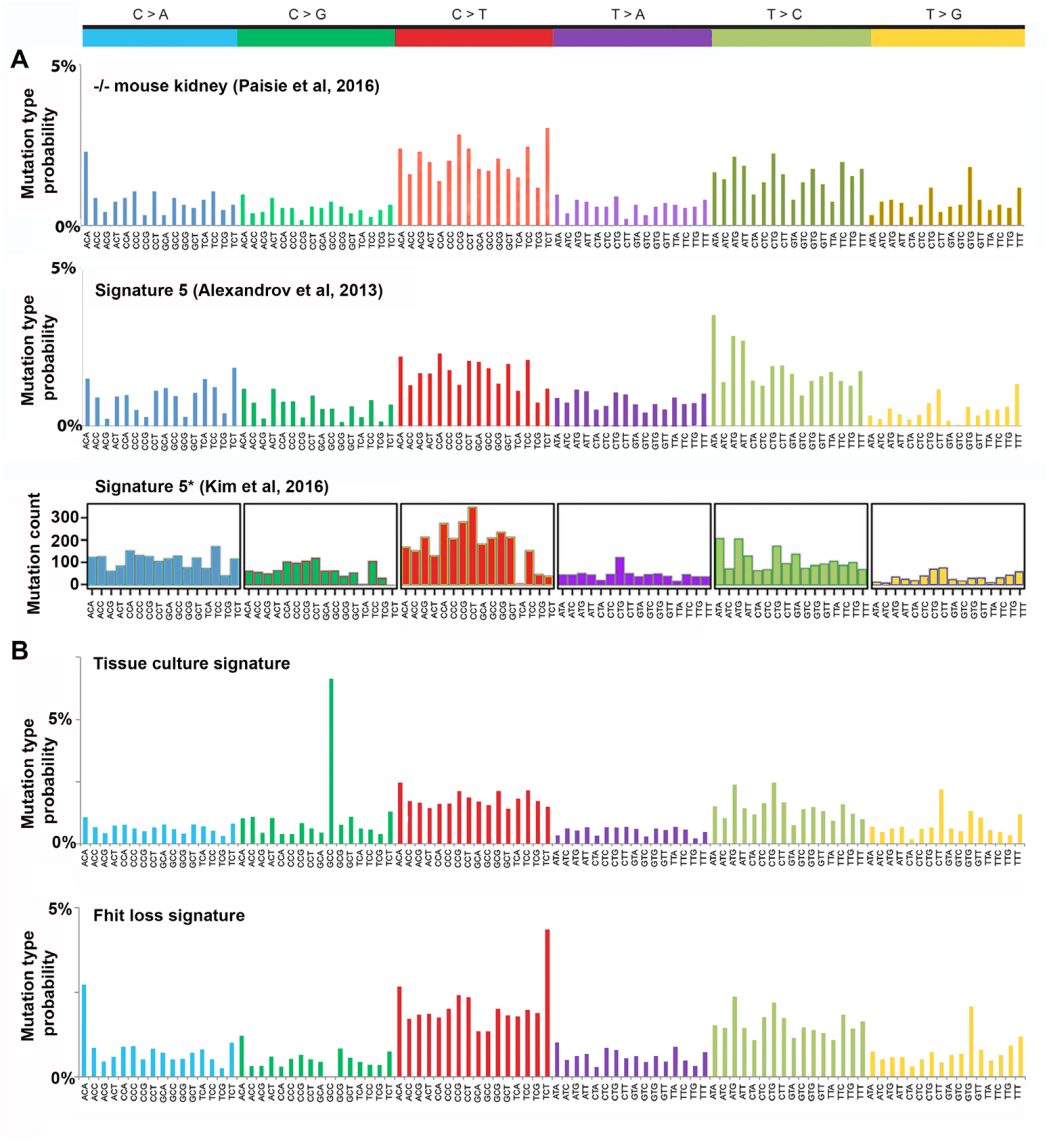
Fhit knockout mutational signature compared to signature 5 profiles **(A)** Previously described mutational signatures from Fhit ko mouse kidney tissue total mutation profile adapted from Figure 3 of Paisie et al [20], human mutational signature 5 of Alexandrov et al [10], signature 5^*^ from Kim et al [21] that used a different algorithm to define mutational signatures. {Permissions: upper panel, Paisie et al (2016) under CC BY-NC 4.0 license (https://creativecommons.org/licenses/by-nc/4.0/legalcode); middle panel, adapted by permission from Macmillan Publishers Ltd: [Nature] Alexandrov et al. 2013; lower panel, adapted by permission from Macmillan Publishers Ltd: [Nature Genetics] Kim et al. 2016}. **(B)** The mutation spectra for *Fhit*-/- lung, kidney tissues, two -/- kidney cell lines, a totipotent and differentiated -/- ESC cell line, were assessed for mutational signatures using the SomaticSignatures algorithm [22]. The upper panel (tissue culture signature) shows a signature occurring primarily in the two kidney cell lines, with a distinctive peak of C to G SBSs at GCC trinucleotides; the lower panel (*FHIT* loss signature) shows a mutational signature dominated by C to T and T to C SBSs that may be the murine equivalent of the human signature 5 shown in panel A.

As noted in Paisie et al [20], the mutation profile in Fhit ko tissues and cell lines is characterized by increased C>T and T>C mutations, resembling human mutational signature 5 [10]; compare Figure 1A, upper and middle panels. Alexandrov et al [10], by 2013, had analyzed >5 million mutations in >7000 cancers, from which they extracted distinct mutational signatures, some of which were observed in many cancer types, including: two signatures, 2 & 13, attributed to the APOBEC family of cytidine deaminases; signature 1 associated with patient age at cancer diagnosis; signatures 4, 7, 11 associated with known mutagenic exposures; signatures 3, 6, 10 associated with mutations in DNA repair proteins, known as genome caretakers. The mutation sources for many signatures, including signature 5, were unknown; however signature 5 (Figure 1A middle panel) was recently shown, along with the 'aging' signature, to be associated with sequences of genomes of all 40 cancer types [10, 11 and COSMIC Database http://cancer.sanger.ac.uk/cosmic], 22 of which also showed APOBEC signatures 2 & 13. Depictions of signature 5 are illustrated in Figure 1A, middle and lower panels to show their similarity to each other. Figure 1A, lower panel, designated Signature 5^*^, was extracted from human bladder cancers by Kim et al [21] using non-negative matrix factorization (NMF) algorithms similar to those described by Alexandrov and colleagues. Despite subtle differences, the *Fhit* ko kidney mutation spectrum has a cosine similarity of 0.85 and 0.80, with COSMIC signature 5 and Kim et al [21] signature 5^*^, respectively. A cosine similarity of 0.00 indicates completely different and a similarity of 1.00 indicates a perfect match.

### Mutational signatures in *Fhit* knockout cells and tissues

To confirm the similarity of the Fhit ko mutation profile to human cancer mutational signature 5, we used the SomaticSignatures package [22] and extracted two mutational signatures from exome sequences of six mouse *Fhit* ko samples described in Paisie et al [20] (Figure 1B). Because most tumors contain more than one mutational signature, the SomaticSignatures package enables users to extract a user-defined number of expected signatures from sequencing data, with power to detect signatures increasing with the number of signatures entered into the algorithm. DNA from age-matched wt mice exhibited a range of 19 to ~300 SBSs, too few to reliably extract signatures, while Figure 1B depicts two signatures (‘Tissue culture signature’ and ‘Fhit loss signature’) that were extracted from six DNA samples obtained from kidney, liver and embryonic stem cells of Fhit -/- mice, all having between 2,596 and 4,757 SBSs; the Fhit ko mouse tissues exhibit elevated numbers of mutations because of absence of the Fhit genome caretaker function [20]. The 'tissue culture signature' found in exome sequences of cells subcultured *in vitro* >15 times [20], features a prominent GCC to GGC mutation. This signature was not found in the exomes of the mouse tissues or embryonic stem cell lines (ESC, subcultured *in vitro* for 1 or 2 passages) examined. The 'Fhit loss mutational signature' occurred in exome DNAs of all *Fhit* ko mouse samples, both cell lines and tissues. This signature closely resembles the reported mouse kidney *FHIT* ko mutation profile shown in Figure 1A, upper panel, and has a cosine similarity value of 0.89 with COSMIC signature 5. For comparison, signature 5 from Alexandrov et al [10] is shown in Figure 1A, middle panel (see Supplementary Figure 1 for the individual fitted spectra from the application of the SomaticSignatures package for total SBS graphs for each tissue and cell line). As a guide for the impact of cosine similarity values, we calculated the values for signatures 2 (0.38, an apobec expression signature), 8 (0.71, a loss of mismatch repair signature) and 13 (0.24, another apobec expression signature) for comparison with the ‘Fhit loss mutational signature’.

### *FHIT* loss correlates with the mutational signature 5 substitution rate in human cancer

To determine whether loss of *FHIT*, or 86 other cancer-relevant or cancer-driver genes [23, 24] (see Supplementary Table 1 for gene list), may be associated with the reported prevalence of mutational signature 5 in human cancers, we compiled the Somatic Copy Number Alterations (SCNAs) of *FHIT* and the other 86 cancer genes in The Cancer Genome Atlas (TCGA) samples (n=6649) previously characterized for mutational signatures. Spearman Rho and robust regression MM estimates [25, 26] were computed between the signature 5 somatic substitution rate per Mb [13] and thresholded GISTIC2 score for the selected SCNAs genes, across the TCGA cohorts. Table 1 lists the genes with SCNAs that were negatively or positively associated with mutational signature 5 in the 6649 cancer samples, identifying *FHIT* as the gene exhibiting the most significant, and negative, correlation with mutational signature 5 (Spearman p= 2.00E-65, MM Regression p= 1.90E-25); i.e., *FHIT* copy loss (negative GISTIC2 scores) was associated with higher density of signature 5 substitutions in the genomes of cancer cells. Supplementary Table 2 shows substitution rate/Mb [12] *vs FHIT* allele copy number (thresholded GISTIC2 score) for mutational signature 5 in each TCGA sample. Other fragile site genes included in this study, such as *PARK2* and *WWOX*, did not correlate with signature 5. *NAALADL2*, the second most significant signature 5-associated gene, exhibited positive correlation, not coherent with its loss leading to higher signature 5 substitutions. A few deleted genes showed significant but weaker negative correlations, viz *CSMD1, RYR2, DSCAM*, while *TP53, ETV6* and *MAP2K4*, like NAALADL2 showed incoherent trends.

**Table 1:** Correlation of SCNA genes with mutational signature 5

Gene Symbol	SpearmanRho	Rhop	MMRegression	MMp
**Deleted genes**				
FHIT	-0.21	2.00E-65	-0.18	1.90E-25
*NAALADL2*	*0.2*	*5.40E-63*	*0.16*	*2.80E-23*
CSMD1	-0.1	2.50E-17	-0.08	1.60E-11
PDE4D	-0.1	9.00E-17	-0.064	2.00E-05
*ETV6*	*0.1*	*1.10E-15*	*0.076*	*2.50E-07*
*MAP2K4*	*0.07*	*3.20E-09*	*0.074*	*2.50E-07*
DSCAM	-0.07	3.30E-09	-0.069	5.00E-06
*TP53*	*0.07*	*6.60E-09*	*0.08*	*3.80E-08*
ERG	-0.07	1.40E-08	-0.064	1.40E-05
RB1	-0.07	8.10E-08	-0.054	2.40E-05
RYR2	-0.05	8.10E-06	-0.058	2.00E-06
*MACROD2*	*0.05*	*1.90E-05*	*0.054*	*1.30E-05*
**Amplified genes**				
CCND1	0.08	1.70E-11	0.092	3.20E-10
MYCN	0.08	4.20E-10	0.079	8.80E-06
CRKL	0.07	2.20E-08	0.063	2.60E-05
*MDM4*	*-0.07*	*2.60E-08*	*-0.067*	*1.30E-07*
MYC	0.06	1.20E-07	0.054	1.20E-05
*MCL1*	*-0.06*	*1.10E-06*	*-0.058*	*5.30E-06*

In this test, genes deleted in cancers significantly associated with mutational signature 5 were expected to show negative correlation, while for amplified genes the opposite trend was expected. Italic font notes genes with incoherent trends.

Bonferroni corrected alpha of 0.05 was 2.8E-05.

Next, we looked for associations between *FHIT* loss and other mutational signatures. The number of TCGA cancers assessed for each cancer type was dependent on the occurrence of individual mutational signatures in specific TCGA cancer types; for each signature, a Spearman correlation and MM regression were computed for the comparison of the somatic substitution rate/Mb *vs FHIT* copy number (Table 2). Only two signatures were significant both by Spearman correlation and robust regression, signatures 5 and 2. Mutational signature 5 was the signature most negatively correlated with *FHIT* copy number alterations, closely followed by mutational signature 2, caused by activity of enzymes A3B or A3A (n=3702, Rho p= 5.30E-43, MM p= 1.1E-21); A3B mutations were previously shown to be increased in cells with Fhit expression loss [9]. Signature 13, the other A3B/A signature, was also correlated with *FHIT* SCNAs, though less significantly and not confirmed by the MM regression test.

**Table 2:** Correlation of *FHIT* loss with mutational signatures in TCGA cohorts

Signature	FHIT loss &substitution rateSpearman Rho	Rho p	N	MM regression	MM p
Sign.5	-0.21	2.00E-65	6649	-0.18	1.9E-25
Sign.2	-0.22	5.30E-43	3702	-0.18	1.1E-21
Sign.7	0.52	4.60E-68	975	ns	ns
Sign.13	-0.11	7.20E-08	2234	ns	ns
Sign.3	-0.10	1.20E-05	1757	ns	ns
Sign.6	0.05	0.001	3629	ns	ns
Sign.26	0.08	0.002	1546	ns	ns
Sign.17	-0.08	0.003	1301	ns	ns
Sign.18	-0.15	0.006	337	ns	ns
Sign.8	-0.06	0.045	974	ns	ns

The table shows the significant correlation values of *FHIT* allele losses (only homozygous deletions were used) with 10 different COSMIC mutational signatures. All TCGA cancer cohorts with available data were analyzed. N is total number of cancer samples exhibiting the specific signature (see Supplementary Table 3 for cancers exhibiting the specific signatures); ns, not significant.

### Genes with mutations or SCNAs associated with *FHIT* allele loss

We also assessed allele losses, gains and gene mutations, for association with loss of *FHIT* in the TCGA cohorts by examining the genes listed in Supplementary Table 1. Deletions (allele loss) were defined by -2 GISTIC2 thresholded score, and amplifications (allele gain) by 1 and 2 GISTIC2 thresholded scores. Low-level hemizygous deletions (GISTIC2 score= -1) were excluded from the analysis, since single copy loss might not lead to an overt molecular phenotype for some SCNAs, due to haplo-sufficiency. The resulting data, in Table 3, shows that genome alterations of 49 cancer-related genes are significantly associated with *FHIT* deletion, i.e., they are lost, gained or mutated when *FHIT* is deleted; 24 genes were often amplified when *FHIT* was deleted, with *KRAS* and *MYC* being the most significant. Sixteen SCNAs were deleted in association with *FHIT*, with *WWOX* as the most significant one. Nine somatically mutated genes (SMGs) were associated with *FHIT* loss, with *TP53* most significant, and *PBRM1* and *VHL* mutations also associated with *FHIT* loss. Among all tested cancer genes, whether deleted, amplified or mutated, *IDH1* was the only one with negative log2 odds ratio, i.e. it was mutually exclusive of *FHIT* deletion, meaning that cancers with significantly mutated *IDH1* genes do not show *FHIT* allele loss.

**Table 3:** Genes altered in association with *FHIT* allele loss

Gene Symbol	Log2 Odds Ratio	Fisher test p
**Somatically mutated genes^a^**		
TP53	1.51	4.00E-12
PBRM1	2.25	1.50E-06
MLL2	1.46	5.50E-06
VHL	2.16	5.90E-06
MLL4	1.56	6.60E-05
SMC3	2.3	7.10E-05
IDH1	-2.16	7.10E-05
CREBBP	1.46	0.00012
LRRK2	1.45	0.00017
**Amplified genes^b^**		
KRAS	1.8	4.50E-14
MYC	1.59	3.90E-13
HMGA2	1.75	2.10E-12
MYCN	1.86	8.20E-12
NCOA3	1.47	1.00E-11
BCL2L1	1.47	1.00E-11
TERT	1.61	1.90E-11
MDM2	1.64	3.00E-11
IGF1R	1.94	5.10E-11
CDK4	1.57	1.60E-10
CCNE1	1.52	1.80E-09
FGFR1	1.5	2.60E-09
CCND1	1.42	1.70E-08
SKP2	1.28	2.00E-07
MYB	1.63	4.50E-06
JUN	1.45	1.30E-05
CDK6	0.94	1.70E-05
EGFR	0.9	3.30E-05
AR	1.19	3.90E-05
LMO1	1.37	7.00E-05
PDGFRA	1.26	0.00035
KIT	1.3	0.00036
BIRC2	1.11	0.00043
YAP1	1.1	0.00044
**Deleted genes^c^**		
WWOX	3.66	1.30E-30
PDE4D	3.67	6.00E-25
PTPRD	3.27	1.60E-21
NAALADL2	4.62	5.70E-19
MACROD2	3.17	1.30E-12
LRP1B	2.96	4.00E-12
PTPRN2	4.11	1.50E-10
PARK2	2.44	7.80E-08
CSMD1	1.9	8.70E-07
ATM	2.92	2.50E-06
DMD	2.37	2.60E-06
CDKN2A	1.35	5.10E-06
PRKG1	2.61	3.20E-05
CNTNAP2	2.8	3.40E-05
DLG2	2.69	0.00035
PARD3B	2.4	0.00041

Log2 OR >0 means that the cancer event was more frequent in tumors with *FHIT* deletion than in those with normal *FHIT*. Log2 OR <0 means the event was mutually exclusive of *FHIT* deletion. Fisher exact test was performed to evaluate association of cancer events. ^a^ Mutated gene score=1, wt score=0. Bonferroni correction for 189 tests (alpha=0.05), p threshold = 0.0003. ^b^ Bonferroni correction for 33 tests (alpha=0.05), p threshold = 0.001. ^c^ Samples that had low-level deletions (GISTIC -1) were not included in this analysis. Bonferroni correction for 45 tests (alpha=0.05), p threshold = 0.001.

### Signature 5 mutations are prominent in cancers with reduced *FHIT* expression

Alexandrov & co-investigators have examined cancer mutational signatures in more detail in specific cancers and noted that base changes in tumor genomes could reveal the causes and paths of cancer evolution [27–29] whether due to known carcinogens or unknown processes. For example, analysis of 41 B-cell lymphoma exomes (Cell Lines Project, The Wellcome Trust Sanger Institute, COSMIC database [11] showed 67 to >9000 mutations comprised of mutational signatures 1, 2, 5 & 9. Mutational signatures 1, 2 & 9 are caused by: aging; APOBEC family enzymes; and Ig gene hypermutation, respectively. However, the most prevalent signature in most lymphomas had an unknown cause, signature 5, which was of interest because B-cell lymphomas occurred spontaneously in *Fhit* ko mice [30]. Gastric, hepatocellular cancers [28], and Acute Myelogenous Leukemias (AMLs) have also shown a high frequency of signature 5 mutations, with AMLs showing nearly exclusively signature 5 [10]. These are all cancers that show large Fhit-deficient fractions [31–33]. In addition, there is evidence for a protective function of Fhit protein in gastrointestinal tumors as oral delivery of a gastrointestinal tract carcinogen, NMBA, caused a 10-fold increase in upper gastrointestinal tumors in young *Fhit* ko mice [34].

**Figure 2:**
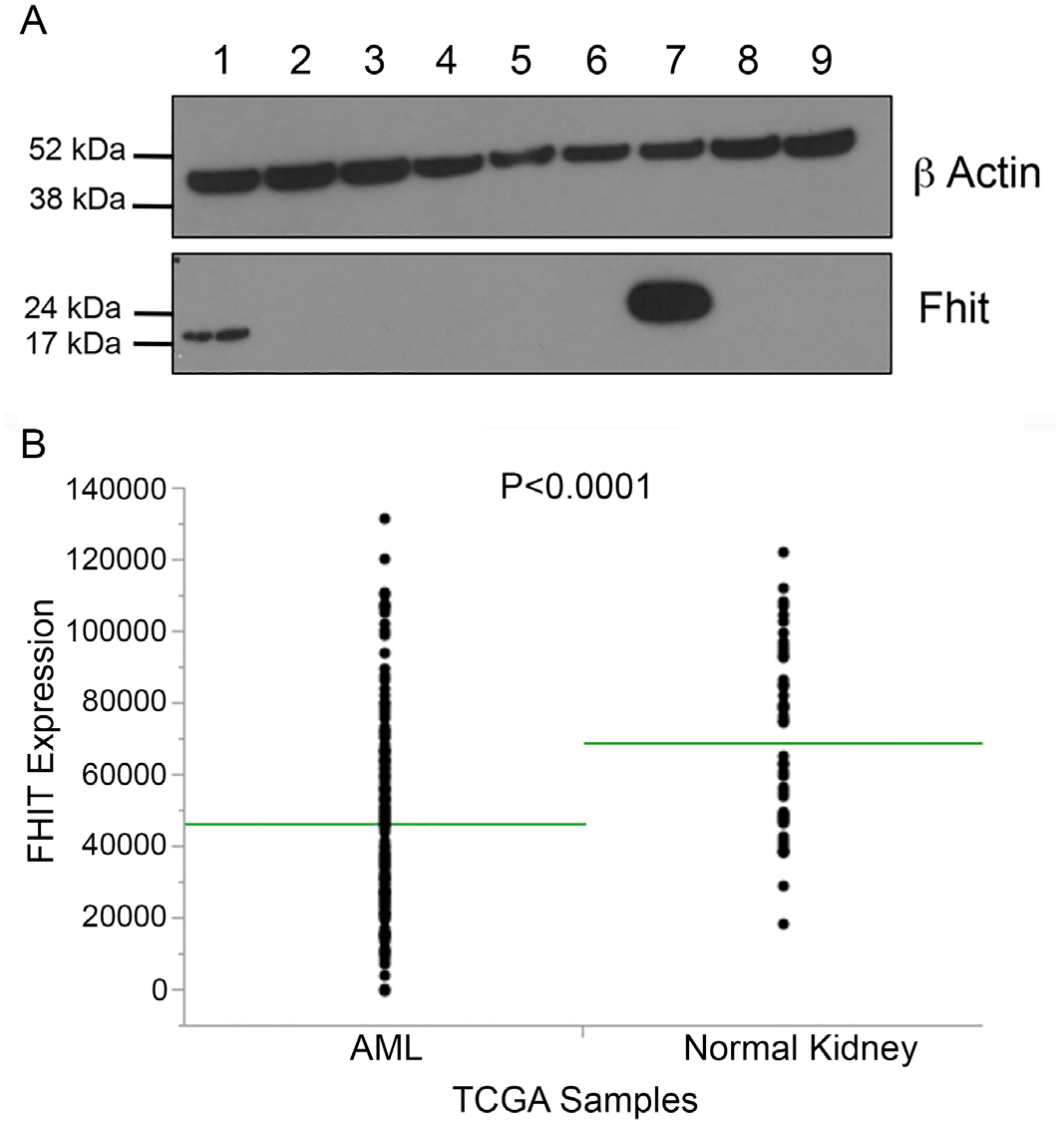
Fhit expression in Acute Myelogenous Leukemias, a cancer exhibiting only mutational signatures 1 and 5 **(A)** Immunoblot depicting loss of Fhit protein expression in 7 of 8 AML bone marrow samples (lanes 1-6, 8 & 9), positive control H1299 D1 cells with induced Fhit expression (lane 7). **(B)** Fhit expression in samples accessed through Genome Data Commons, depicting reduced *FHIT* RNA expression in 153 AMLs derived from bone marrow *vs* 50 kidney samples derived from normal tissue. Neither normal bone marrow nor matched peripheral white blood cell RNA was available for comparison.

To show examples of absence of Fhit protein expression in cancers where signature 5 is the predominant mutational signature, we performed immunoblots for Fhit protein using lysates of 8 AMLs. One of the AMLs expressed some Fhit protein, likely due to <50% blasts in the cell sample, while the others were negative or had very reduced Fhit protein expression (Figure 2A). Additionally, we examined the TCGA database for AMLs with mRNA expression data and found that *FHIT* mRNA expression in 153 human bone marrow-derived AML samples accessed through the Genomic Data Commons portal [35], is significantly reduced relative to 50 normal kidney tissue samples (Figure 2B).

## DISCUSSION

**Figure 3:**
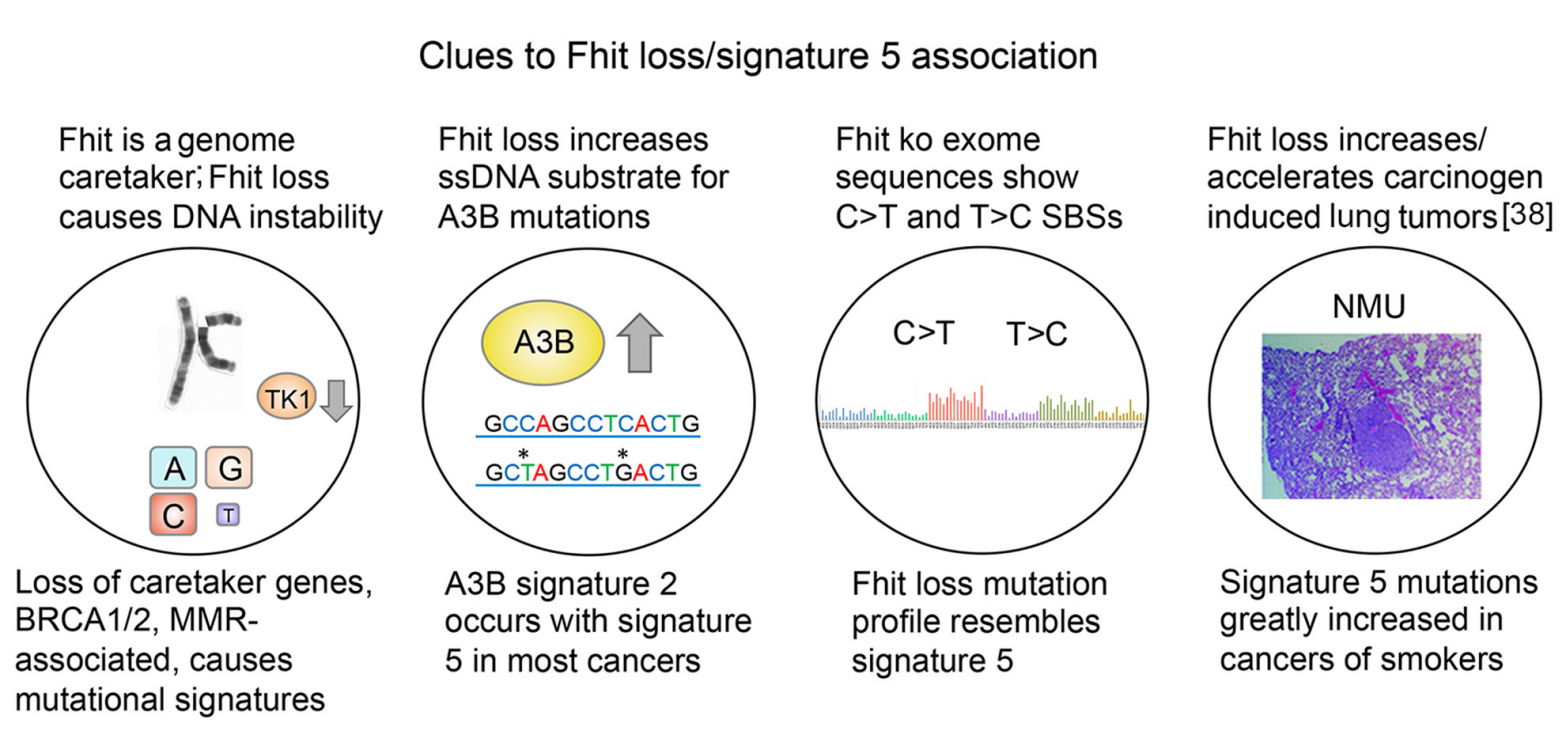
Features of cells with *FHIT* loss (above circles), and specific mutational signatures (below circles)

Previous findings that led to our proposal that *FHIT* loss causes signature 5 mutations were: *FHIT* loss and signature 5 mutations occur in most types of cancer; *FHIT* loss and signature 5 mutations occur early in the neoplastic process; signature 5 mutations are age-associated and the expectation is that frequency of *FHIT* loss in a given cancer type should be age-associated due to fragile site breakage throughout life [36, 37]; loss of *FHIT* causes genome instability [7] and might, like loss/mutation of other caretaker genes, cause specific mutational signatures; the mutation profile of the *Fhit* ko mouse cells and tissues closely resembled mutational signature 5 (see Figure 3 model).

In confirmation of this proposal, we have shown that the Fhit-loss signature extracted from the exomes of ko mice has a cosine similarity of 0.89 with COSMIC signature 5. In addition, data mining of over 6,500 TCGA cancers identified *FHIT* as the gene exhibiting the most significant, and negative, correlation with mutational signature 5 (Spearman p= 2.00E-65, MM Regression p= 1.90E-25). SCNAs in other fragile genes do not correlate with Signature 5. Confirming this specificity, we found that Fhit loss correlated only with Signature 5 and Signature 2, the A3B signature, as expected given that Fhit loss generates ssDNA substrates for A3B hypermutation [9]. Lastly, we identify other cancer-associated genes that negatively or positively correlate with Fhit loss.

**Figure 4:**
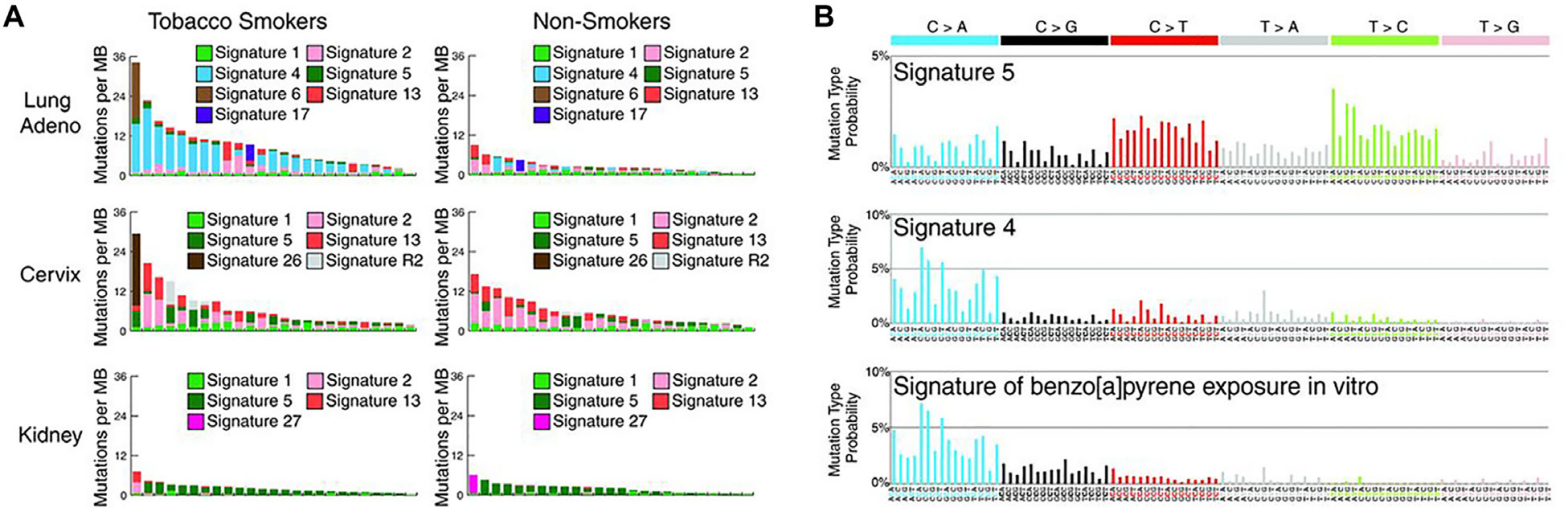
The mutational signatures in smokers *vs* nonsmokers An abbreviated copy of the “smoking signatures” from Figure 2 of ref 39 {from Alexandrov LB et al. 2016. Mutational signatures associated with tobacco smoking in human cancer. *Science* 354: 618-622. Reprinted with permission from AAAS}, to emphasize features of this signature that make *FHIT* loss a strong candidate as cause of mutational signature 5: **(A)** illustration of the mutation spectra in 25 randomly selected cancer genomes (individual bars from smokers or nonsmokers of a given cancer type). Each bar is colored proportionately to the number of mutations/Mb of the specific mutational signatures found in the sample genome. **(B)** The pattern of mutational signatures observed in tobacco smoker cancers.

A telling study, also in accord with the strong association of *FHIT* loss with mutational signature 5, was the recent report of the mutational consequences of smoking [39], comparing somatic mutations in smokers *vs* nonsmokers for lung and other cancers known to be increased in cigarette smokers (Figure 4, examples from Figure 2 of ref 39). Increases in smokers *vs* nonsmokers were reported for signatures 2 & 13 (APOBEC), signature 4 (C>A mutations due to tobacco smoke carcinogens), 5 & 16 (origins unknown). These mutation increases were apparently of clonal mutations for signatures 4 and 5 [39], due to smoke exposure during preneoplastic stages. In Figure 4A, mutational signature 5 showed mutations across all 96 mutation subtypes, with more T>C and C>T mutations, similar to the signature of *Fhit* ko tissues (Figure 1). Signature 5 mutations were found in all cancer types studied in this report [39], including the cancers of the nonsmokers, as expected for cancers with *FHIT* loss. Signature 5 mutations were increased in smokers *vs* nonsmokers in lung, larynx, pharynx, oral cavity, esophageal squamous, bladder, liver, cervical and kidney cancers [39] in a non-age related manner, since carcinogens in cigarette smoke would have compounded the signature 5/*FHIT* loss signature, as noted in the Figure 3 model. These are all cancers for which reduced Fhit expression has been reported [2, 40–45]. In considering Figure 4, there are striking findings consistent with a role for *FHIT* loss in production of signature 5 mutations. First, these alterations can occur early in the preneoplastic process and would appear as clonal alterations in a tumor, just as alterations within the fragile *FHIT* locus are clonal in cancers and cancer cell lines [1, 46, 47]. *FHIT* alterations and loss of expression occur more frequently in precancerous lung tissues of smokers [2, 40, 41, 48], such that lung adenocarcinomas would show *FHIT* loss unrelated to age at diagnosis. We also know from the study of Waters et al [9], that loss of *FHIT* expression creates optimal ssDNA substrates for A3B enzyme activity, and in many smoker and nonsmoker cancers of the cervix, mutational signature 5 occurs in the same tumors as APOBEC signature 2 & 13 mutations (Figure 4A panels). This is also true in the oral cavity and bladder cancers of smokers [39], all cancers for which Fhit protein expression is lost or reduced in large fractions of cases. The kidney cancer results (ref 39 & Figure 4A) where the mutation burdens are low in smoking associated and non-associated cancers, with signature 5 as the main signature observed (Figure 4), is satisfying at the genetic level since the *FHIT* gene was originally cloned from cells of an individual at high risk for multifocal, bilateral clear cell kidney cancer that occurred in family members carrying an inherited, balanced chromosome translocation between chromosomes 3 & 8, where the chromosome 3 translocation break was within the fragile FRA3B locus, interrupting the *FHIT* gene [1].

As for the originating mechanism for the Fhit loss signature, and theoretically for signature 5, it may arise partly because of the down-regulation of TK1 expression by loss of Fhit expression and subsequent genome instability [7]. The signatures compiled from exome sequences of *Fhit* ko kidney and lung tissue and ko kidney cell lines revealed accumulation of >2000 SBSs per exome sequence and increased numbers of C>T and T>C SBSs, with a signature highly similar to mutational signature 5. As noted previously [21], C>T transitions may be generated through spontaneous deamination or DNA replication errors at CpG dinucleotides. SBS peaks for ACG, CCG, GCG, & TCG sequences, where the central C transitions to T, include such dinucleotide sequences. The T>C transitions observed in *Fhit* ko tissues may be due to the increased ratio of dUTP:TTP, allowing misincorporation of dUTP in place of TTP. Depending on the involved translesion polymerase, DNA replication might insert a guanine or a cytosine across from abasic sites and after another round of DNA replication, will result in a T>C or a T>G SBS [7, 20, 49–51].

Kim et al [21] have previously examined the mutational processes in urothelial cancer, a cancer in which the NER gene ERCC2 is significantly mutated, and proposed that such tumor cohorts demonstrate a strong association between somatic ERCC2 mutations and mutational signature 5. These investigators noted an association of signature 5 mutations and smoking that was not associated with ERCC2 mutation status, while there is strong evidence that *FHIT* loss is associated with exposure to cigarette smoke [2, 40, 48]. Since Fhit is a genome caretaker protein, it is possible that its loss, which occurs frequently in bladder cancer [45], could predispose to the *ERCC2* mutations associated with these cancers.

Tomasetti & Vogelstein [52, 53] have recently reported that 'Variation in cancer risk among tissues can be explained by the number of stem cell divisions', and that these mutations are responsible for 2/3 of the mutations in human cancers. The authors emphasize the importance of early detection and intervention to reduce disease and death for the cancers arising from 'unavoidable' mutations that are a result of errors during replication stress. We have presented data here that supports the hypothesis that *FHIT* loss is the underlying determinant of mutational signature 5, a signature that is ubiquitous in human cancers. We propose that many of the ‘unavoidable’ mutations in cancer, such as those significantly associated with *FHIT* loss in Table 3, are due to the genome instability introduced through *FHIT* allele alterations caused by replicative stress at the FRA3B locus. If replicative stress at this fragile site or the results of such stress could be prevented, many 'unavoidable' mutations might be avoided.

Most cancer research is currently focused on finding targets for curing patients with progressive or advanced cancer; thus these studies are looking for cancer driver genes, which may be targeted by specific drugs. At the same time, investigators should be considering prevention of new cancers. As noted above, Tomassetti & Vogelstein [52, 53] have proposed that 2/3 of mutations in cancer may be unavoidable, a result of errors during replication. Many of these ‘unavoidable’ mutations, signature 5 mutations, likely associated with replication stress occurring during and after *FHIT* allele alteration, may in fact be avoidable if we can target the genome instability associated with *FHIT* loss, for example by low dose thymidine supplementation. At the very least, onset of preneoplasia in at-risk tissues might be predicted by following *FHIT* gene alterations by RT-qPCR [1]. With very early detection, such cancers would be more easily cured.

## MATERIALS AND METHODS

### Data mining, computational methods and statistics

Publicly available mutational signatures were obtained for all TCGA samples examined for mutational signatures, using the Wellcome Trust Sanger Institute algorithm by Alexandrov et al [12]. The molecular portraits of TCGA samples, including somatic mutations and copy number alterations (SCNA as scored by GISTIC2; Genomic Identification of Significant Targets in Cancer, version 2.0) were obtained from Firebrowse (http://firebrowse.org). The list of the frequent SCNAs in cancer were obtained from the SCNA list of Beroukhim et al [23] and completed with the deleted and amplified cancer driver genes described by Vogelstein et al [24]. See Supplementary Table 1 for listing of the 87 total genes, including *FHIT*, 44 deleted, 31 amplified and 12 mutated.

The thresholded GISTIC2 scores for the SCNA cancer genes obtained from FireBrowse were investigated for association with mutational signatures, using Spearman correlation and MM regression. Highly stringent Bonferroni correction was implemented, dividing the critical P value (α=0.05) by the number of comparisons being made (i.e. 87 SCNA genes and 24 signatures).

Spearman correlation and MM robust regression (Robustbase package in R; this function computes an MM-type regression estimator as described [25, 26] were computed for lists generated in Tables 1, 2 and 3. We chose DNA copy number variation as the most robust method to establish a link between *FHIT* and cancer signatures. *FHIT* does not exhibit somatic mutations and Fhit protein expression data are not available in large-scale cancer studies, such as in TCGA.

### Tissues, cell lines and exome sequences

The *Fhit*+/+ and -/- tissues and cell lines from the constitutive ko strain have been described, as have the exome sequence results and analyses [20]. Females of the constitutive *Fhit^-/-^* strain and our laboratory B6 strain, were superovulated by hormone treatment, mated and embryos collected for isolation of inner cell mass cells and establishment, characterization and freezing of embryonic stem cell lines. The ESC cells were grown briefly in stem cell culture conditions before isolating DNA for exome sequencing; some ESC cells were then switched to non stem cell medium and allowed to differentiate through several subcultures before preparing DNA for sequencing. The exome sequencing data for the ESCs has been added to SRA BioProject PRJNA260539 (will be released on or before publication of this manuscript), where sequence files for the other Fhit ko samples are already available.

Filtered SBS numbers in wt DNAs ranged between 19 and ~300, numbers too low to reliably examine in the SomaticSignatures algorithm [22]. Whole exome sequences of the following tissues and cell lines exhibited >2500 SBSs each and were analyzed for mutational signatures as described [10, 11, 22]: ko kidney tissue, 2912 SBSs; ko lung tissue, 2596; NS1 ko kidney cell line, 4631; NS4 ko kidney cell line, 4757; ko ESC line, 3521; ko ESC derived differentiated line, 3028. Mutational signatures were derived from these six samples using the SomaticSignatures R package [22] and R version 3.2.4 for Windows. Mutation spectra were decomposed using NMF [54]; signature decomposition was determined for 2, 3, or 4 signatures. Manual examination of signatures, and comparison with previously published signatures [12, 54], was used to determine that two was the number of signatures readily noted in the *Fhit* ko exome sequences. The SomaticSignatures package [22] was used to generate mutational signatures from the six Fhit ko samples described above. The matrix and NMF_fitted_Spectrum generated by SomaticSignatures is shown in Supplementary Table 4 and Supplementary Figure 1, respectively. Information for the use of SomaticSignatures was found at https://bioconductor.org/packages/release/bioc/vignettes/SomaticSignatures/inst/doc/SomaticSignatures-vignette.html.

### Fhit expression analyses in cancers

Immunoblotting was performed as described using anti-beta actin (sc-1616) and polyclonal rabbit anti-Fhit serum [7]. The deidentified human AML samples were obtained from the OSUCCC leukemia tissue bank. For *FHIT* RNA expression analysis, expression files for 153 bone marrow-derived AML samples and 50 normal kidney samples were chosen at random and the Fragments Per Kilobase of transcript per million mapped reads files, normalized to upper quartile (FPKM-UQ), were downloaded from Genome Data Commons [35]. The expression values were plotted in JMP and a non-parametric Wilcoxon two-sample test was performed (depicted in Figure 2B).

## SUPPLEMENTARY MATERIALS FIGURES AND TABLES







## Abbreviations

A3B: APOBEC3B
COSMIC: Catalog of Somatic Mutations in Cancer
SBS: single base substitution
CNAs: copy number alterations
wt: wild-type
ko: knockout
NMF: non-negative matrix factorization
ESC: embryonic stem cell
SCNAs: Somatic Copy Number Alterations
TCGA: The Cancer Genome Atlas
SMGs: somatically mutated genes
AMLs: Acute Myelogenous Leukemias
