# Supplementary material for: The ubiquitous ‘cancer mutational signature’ 5 occurs specifically in cancers with deleted FHIT alleles
Source: Oncotarget. 2017 Nov 6;8(60):102199–211. doi: 10.18632/oncotarget.22321 (PMC5731946; doi:10.18632/oncotarget.22321)
Supplement: Supplementary file 1 [file oncotarget-08-102199-s001.pdf]

The ubiquitous 'cancer mutational signature' 5 occurs specifically in cancers with deleted *FHIT* alleles

SUPPLEMENTARY MATERIALS

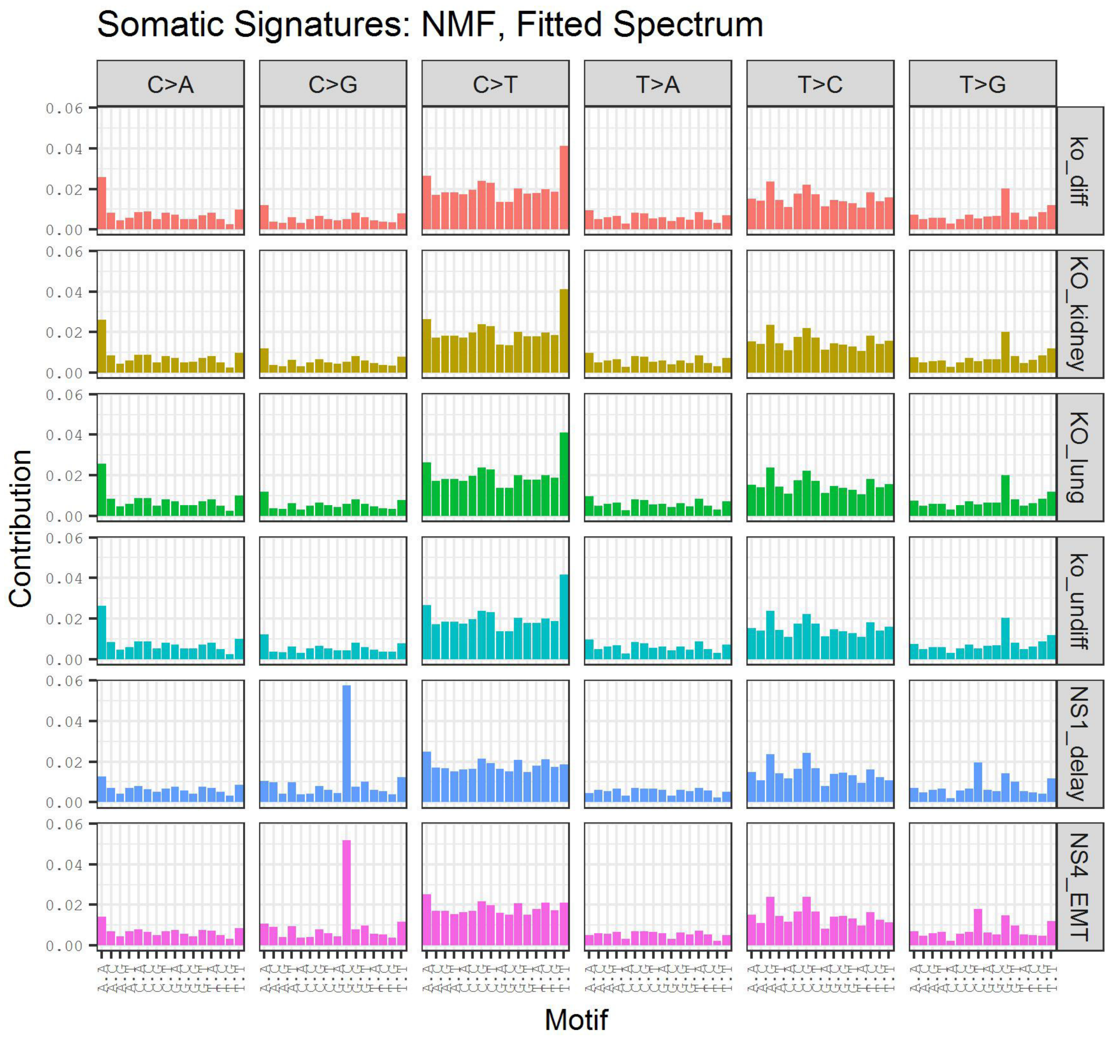

Supplementary Figure 1: SomaticSignatures: NMF, Fitted Spectrum.

Supplementary Table 1: Table of all genes used in these analyses

See Supplementary File 1

Supplementary Table 2: COSMIC signature 5 substitution rate vs. *FHIT* copy number (GISTIC2 thresholded)

FHIT copy number (GISTIC2)	Signature 5 density (median)	N
Homozygous deletion (-2)	1.00	196
Heterozygous deletion (-1)	0.93	2360
Normal (0)	0.43	3517
Low level Amp (1)	0.43	551
High level Amp(2)	0.97	25

Supplementary Table 3: Table of cohort types and numbers used in Table 2 analyses

	Number of Cases	Cohort (number of cases)
Signature 2	3702	KIPAN (169), LUSC (176), THCA (401), STES (520), BLCA (235), BLCA (235), HNSC (505), LUAD (487), BRCA (974), UCEC (235)
Signature 5	6649	KIPAN (682), SKCM (294), UCEC (235), LUSC (176), THCA (401), LAML (186), GBMLGG (733), COADREAD (480), BLCA (235), HNSC (505), PRAD (295), LUAD (487), BRCA (974), OV (446), STES (520)
Signature 18	337	STES (337)
Signature 13	2234	BRCA (974), STES (520), BLCA (235), HNSC (505)
Signature 3	1757	BRCA (974), STES (337), OV (446)
Signature 17	1301	STES (520), SKCM (294), LUAD (487)
Signature 8	974	BRCA (974)
Signature 6	3629	KIPAN (513), STES (183), GBMLGG (462), COADREAD (480), UCEC (235), PRAD (295), LUAD (487), BRCA (974)
Signature 26	1546	BRCA (974), STES (337), UCEC (235)
Signature 7	975	HNSC (505), SKCM (294), LUSC (176)

BLCA, bladder urothelial carcinoma; BRCA, breast invasive carcinoma; COADREAD, colorectal adenocarcinoma; GBMLGG, glioma; HNSC, head and neck squamous cell carcinoma; LUAD, lung adenocarcinoma; LUSC, lung squamous cell carcinoma; SKCM, skin cutaneous melanoma; KIPAN, pan kidney; OV, ovarian; STES, stomach and esophageal; UCEC, uterine corpus endometrial carcinoma; THCA, thyroid carcinoma; LAML, acute myeloid leukemia; PRAD, prostate adenocarcinoma.

Supplementary Table 4: m Input matrix 'm'

See Supplementary File 2
