# Supplementary material for: The ubiquitous ‘cancer mutational signature’ 5 occurs specifically in cancers with deleted FHIT alleles
Source: Oncotarget. 2017 Nov 6;8(60):102199–211. doi: 10.18632/oncotarget.22321 (PMC5731946; doi:10.18632/oncotarget.22321)
Supplement: Supplementary file 2 [file oncotarget-08-102199-s002.docx]

**Supplementary Table S1. Table of all genes used in these analyses**

| Beroukhim^a^ Amplified genes | Beroukhim^a^ Deleted genes | Vogelstein^b,c^  genes |
| --- | --- | --- |
| *MYC* | *CDKN2A* | *CDKN2C* |
| *CCND1* | *CDKN2B* | *IKZF1* |
| *ERBB2* | *FHIT* | *LMO1* |
| *CDK4* | *WWOX* | *NCOA3* |
| *NKX2-1* | *PTPRD* | *SKP2* |
| *MDM2* | *MACROD2* | *MLL4* |
| *EGFR* | *PARK2* | *IDH1* |
| *MCL1* | *RB1* | *PBRM1* |
| *FGFR1* | *LRP1B* | *MLL2* |
| *KRAS* | *PDE4D* | *VHL* |
| *CCNE1* | *A2BP1* | *CREBBP* |
| *CRKL* | *TRB* | *SMC3^d^* |
| *HMGA2* | *PTEN* |  |
| *TERT* | *CSMD1* |  |
| *PRKCI* | *DMD* |  |
| *IGF1R* | *OPCML* |  |
| *MYCL1* | *HNT* |  |
| *MYCN* | *ETV6* |  |
| *CDK6* | *NF1* |  |
| *BCL2L1* | *ATM* |  |
| *MYB* | *PRKG1* |  |
| *MET* | *PAX5* |  |
| *JUN* | *TP53* |  |
| *BIRC2* | *PTPRN2* |  |
| *YAP1* | *APC* |  |
| *PDGFRA* | *NEGR1* |  |
| *KIT* | *GPC6* |  |
| *PIK3CA* | *RYR2* |  |
| *MDM4* | *MAGI2* |  |
| *AR* | *CNTNAP2* |  |
| *LRRK2/DCLK1^e^* | *NAALADL2* |  |
|  | *ANKS1B* |  |
|  | *PARD3B* |  |
|  | *SNTG1* |  |
|  | *CDH13* |  |
|  | *DLG2* |  |
|  | *SDK1* |  |
|  | *MAP2K4* |  |
|  | *DSCAM* |  |
|  | *TMPRSS2* |  |
|  | *ERG* |  |
|  | *SMAD4* |  |
|  | *DCC*  *PRSS1* |  |

^a^Beroukhim R et al. The landscape of somatic copy-number alteration across human cancers. Nature. 2010; 463:899-905.

^b^Vogelstein B et al. Cancer genome landscapes. Science. 2013; 33:1546-58. Review

^c^There are overlaps among the lists with some genes being amplified, lost and/or mutated in some cancer cohorts

^d^Shiba N et al. Whole-exome sequencing reveals the spectrum of gene mutations and the clonal evolution patterns in paediatric acute myeloid leukaemia. Br J Haematol. 2016; 175:476-489.

^e^Kadletz L et al. Role of cancer stem-cell marker doublecortin-like kinase 1 in head and neck squamous cell carcinoma. Oral Oncol. 2017; 67:109-118.
